# Transcriptomic and Physiological Analysis Reveals the Responses to Auxin and Abscisic Acid Accumulation During *Vaccinium corymbosum* Flower Bud and Fruit Development

**DOI:** 10.3389/fpls.2022.818233

**Published:** 2022-02-15

**Authors:** Liangmiao Liu, Yiqi Zheng, Shiji Feng, Lei Yu, Yongqiang Li, Yu Zong, Wenrong Chen, Fanglei Liao, Li Yang, Weidong Guo

**Affiliations:** ^1^College of Chemistry and Life Sciences, Zhejiang Normal University, Zhejiang, China; ^2^Zhejiang Provincial Key Laboratory of Biotechnology on Specialty Economic Plants, Zhejiang Normal University, Zhejiang, China

**Keywords:** blueberry (*Vaccinium corymbosum*), flower and fruit development, cell division and expansion, auxin, ABA

## Abstract

Blueberry (*Vaccinium corymbosum*) is reputed as a rich source of health-promoting phytonutrients, which contributes to its burgeoning consumer demand and production. However, blueberries are much smaller and have lower yields than most domesticated berries, and the inherent regulatory mechanisms remain elusive. In this study, the cytological and physiological changes, as well as comparative transcriptomic analysis throughout flower and fruit development in the southern highbush blueberry cultivar ‘O’Neal’ were performed. ‘O’Neal’ hypanthium and fruit exhibited a distinctive cell proliferation pattern, and auxin accumulation was unusual throughout development, while abscisic acid (ABA) levels rapidly increased in association with anthocyanin accumulation, total phenolic reduction and fruit maturation. Transcriptomic data showed that many differentially expressed genes (DEGs) were specifically expressed at each flower bud and fruit developmental stage. Further weighted gene co-expression network analysis (WGCNA) revealed numerous DEGs that correlated with the cell numbers of outer mesocarp and columella, showed two distinctive expression patterns. Most of the DEGs involved in auxin biosynthesis, transportation and signal transduction were upregulated, and this upregulation was accompanied by cell expansion, and flower bud and fruit development. However, individual members of *VcSAUR50* and *VcIAA9* families might be insensitive to auxin, suggesting that these genes play a distinctive role in the growth and development of blueberry fruits. These results will support future research to better understand the flower and fruit development of southern highbush blueberry.

## Introduction

Fruits are unique reproductive structures of angiosperms that promote seed dispersal, and provide natural staple diets and nutrient resources for human beings. Hence, fruit size, quality and morphology are the most important characteristics and yield traits, but these characteristics and traits are undoubtedly mainly affected and regulated throughout flower and fruit development. In fact, the development of flowers and fruits, including flower bud differentiation and enlargement, as well as fruit growth and maturation, is a sophisticated process involving in numerous cytological, physiological, biochemical and molecular changes and is also controlled concomitantly by basic and secondary metabolic pathways, cell cycle, epigenetic modifications, and phytohormones, *etc.* (Bertin et al., 2003; Giovannoni, 2004; van der Knaap and Ostergaard, 2018; Tang et al., 2020; Fenn and Giovannoni, 2021).

Phytohormones are small chemical messengers first identified in the early 20th century that are now known to be active in very low quantities and to function diversely in almost all aspects of a plant’s life cycle, such as cellular activities, organogenesis, reproduction, and responses to abiotic and biotic stresses (Williams, 2010). As described above, flower and fruit development is a sophisticated process, and phytohormones synergistically regulate most stages of flower and fruit development, from flower bud differentiation to fruit maturation. Moreover, precise spatial and temporal control of phytohormone [*e.g.*, auxin and abscisic acid (ABA)] biosynthesis, transport, signal perception and transduction is required for flower and fruit development (Krouk et al., 2011; McAtee et al., 2013; Kumar et al., 2014; Iqbal et al., 2017; Liao et al., 2018; Fenn and Giovannoni, 2021). Auxin, firstly purified in the 1930s, is a plant growth-promoting substance, which plays pivotal roles in parthenocarpy, pollination, fertilization, fruit growth and maturation (Pattison et al., 2014; Fenn and Giovannoni, 2021). Zifkin et al. (2012) also identified auxin is the relevant hormonal signal involved in blueberry fruit maturation. Similarly, ABA is recognized as a major positive regulator and accelerator for fruit maturation and anthocyanin biosynthesis, such as bilberry and blueberry (Karppinen et al., 2018, 2021). Discovering the regulatory interactions between phytohormones and flower bud and fruit development will help us further understand the inherent mechanisms underlying blueberry fruit size and yield.

Blueberry (*Vaccinium corymbosum*), the second most abundant berry in the world after strawberry, is rich in health-promoting phytonutrients, such as anthocyanins (flavonoids), polyphenols (non-flavonoids) and ascorbic acid, which makes the fruit attractive to consumers and cultivators (Skrovankova et al., 2015; Silva et al., 2020). Blueberry belongs to the genus *Vaccinium* which consists of approximately 450 species (Song, 2015). The predominant cultivated blueberry species mainly originated from highbush blueberry (*V. corymbosum*), rabbiteye blueberry (*V. ashei*), lowbush blueberry (*V. angustifolium*) and, hybrids of highbush × lowbush blueberry, while highbush species are further separated into southern and northern types depending on their chilling requirement and winter hardiness (Retamales and Hancock, 2018). Due to its elite cultivars, high quality and increasing consumer demand, southern highbush blueberry is expanding throughout the world (Retamales and Hancock, 2018; Fang et al., 2020). Uncovering the flower and fruit developmental processes of southern highbush blueberry will provide and highlight insights relevant to fruit set, growth, and maturation, as well as fruit size, quality and production.

The aim of this study was to characterize the cytological, physiological and transcriptomic changes throughout southern highbush blueberry cultivar ‘O’Neal’ hypanthium (receptacle and inferior tissues of flower bud, and form fruit after pollination and fertilization; Konarska, 2015) and fruit ontogeny. Particular attention was also given to phytohormones (especially auxin and ABA) biosynthesis, transportation and signal transduction associated with cell proliferation, expansion and fruit development. These results will support future research to better understand southern highbush blueberry flower and fruit development.

## Materials and Methods

### Plant Materials

**FIGURE 1:**
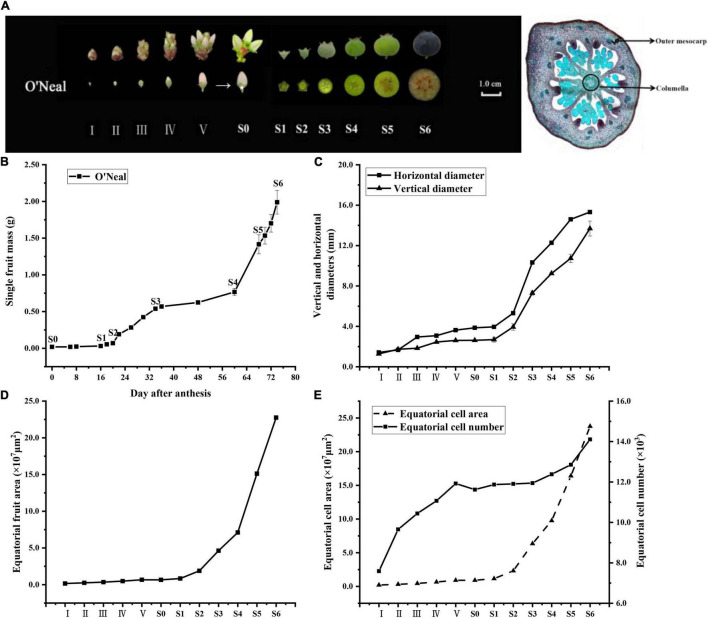
Developmental stages of *V. corymbosum* ‘O’Neal’ flower bud and fruit. **(A)** The floral and berry development stages used in this study, and microscopic images of equatorial hypanthium at stage III (40 × magnification). **(B)** ‘O’Neal’ fruit growth curve. Error bars represented the SD of 60 flower buds and fruits. **(C)** Equatorial hypanthium/fruit dimensions (width and length) and **(D)** equatorial pericarp area at different developmental stages. Error bars represented the SD of three replicates (20 hypanthia and fruits at different developmental stages were used for each replicate). **(E)** Cell numbers and cell area of equatorial hypanthium/fruit sections in different developmental stages. Error bars represented the SD of 3∼5 hypanthia and fruits. Hypanthium (the white asterisk location in the **A**) is the receptacle and inferior tissues of flower bud without stamens, pistil and petals and form fruit after pollination and fertilization. Outer mesocarp is the region between the hypodermis and the first large vascular bundle, and columella is the central axis of fruit.

Sampling was performed in 2019 and 2020 on 8 (or 9)-year-old southern highbush blueberry (*V. corymbosum cv.* ‘O’Neal’) plants located in Jinhua, China (119°65′ E and 29°08′ N), where its chilling time was approximate 394 h (< 7.2°C) in 2020. Flower buds and fruits at specific developmental stages (Figure 1A) were randomly tagged, collected and processed as described previously (Yang et al., 2018). Except those used for paraffin sectioning and physiological analysis, the processed samples were frozen immediately in liquid nitrogen and stored at −80°C for further use.

### Paraffin Section Preparation and Cytological Analysis

Three to five hypanthia and fruits at each developmental stage were prepared for paraffin section according to our previous study (Yang et al., 2018). Equatorial sections ranged from 8∼25 μm thick were stained with safranin and Fast Green, and then imaged on a Virtual Slide Microscopy VS120 system (Olympus Co., Ltd., Shanghai, China). Total cell numbers were determined manually and by ImageJ software, and the average cell area was calculated by dividing the equatorial fruit area by cell number.

### Quantification of Chlorophyll, Phenolics, Anthocyanin, Auxin, and ABA

Hypanthia (30 mg) and fruits (0.5 g) at each developmental stage were extracted in 80% acetone solution, and total chlorophyll contents [Chl (a + b)] were determined according to Arnon (1949). The total contents of phenolic compounds and anthocyanins were extracted and determined according to the protocols described by Lin et al. (2018). Indole-3-acetic acid (IAA) and ABA were extracted, separated and analyzed as described previously (Zhao et al., 2019). All experiments were conducted with at least three biological replicates.

### RNA Sequencing (RNA-Seq) and Bioinformatic Analysis

‘O’Neal’ hypanthia and fruits at specific developmental stage (stages ONIII, ONIV, ONS0, ONS1, ONS2, ONS4, ONS5, and ONS6, respectively) with three biological replicates in 2019 were chosen for RNA-seq (Novogene Science and Technology Co., Ltd., Beijing, China) and bioinformatic analysis (Origingene Biomedical Technology Co., Ltd., Shanghai, China). In brief, the reads with adapters, poly-N (> 10%) or low quality were first removed, and the retained reads were aligned to the reference genome of highbush blueberry *cv.* “Draper” (Colle et al., 2019) by using HISAT2 software (v2.1.0, Kim et al., 2015). Transcript abundances of target genes were expressed as fragments per kilobase per million fragments (FPKM values, Mortazavi et al., 2008).

Principal component analysis (PCA) was performed in the R package and plotted with the scatterplot3d library (Ligges and Maechler, 2003). Differentially expressed genes (DEGs, fold change ≥ 3 and Padj. < 0.01) in each comparison were chosen with DEGSeq2 and StringTie softwares in the R package (Benjamini and Yekutieli, 2001; Andino et al., 2016), and then subjected to enrichment analysis with the Kyoto Encyclopedia of Genes and Genomes Ortholog database (KEGG) and Gene Ontology (GO) database. A Venn diagram of the DEGs was generated by online software^1^. A heatmap was drawn by TBtools software (v1.05, Chen et al., 2020). Gene co-expression networks linked to cell numbers of ‘O’Neal’ outer mesocarp and columella (central axis of fruit, Figure 1A) were constructed by using weighted gene co-expression network analysis (WGCNA) 1.47 software in the R package (Langfelder and Horvath, 2008), and were drawn using Cytoscape 3.6.1 software (Shannon et al., 2003).

### mRNA Transcript Abundance Validation

Total RNA extraction and 1st-strand cDNA synthesis of samples at each developmental stage were performed according to Yang et al. (2018). Real-time quantitative reverse transcription PCR (qPCR) analysis was performed on three biological replicates and in four technical triplicates as described previously (Liu et al., 2016). The relative abundances of target transcripts were normalized to *VcGAPDH* gene (Die and Rowland, 2013) using the comparative cycle threshold (2^–^^ΔΔCT^) method (Livak and Schmittgen, 2001). The specific primer sequences for used qPCR in this study were listed in Supplementary Table 1.

### Statistical Analysis

Data analysis was performed using SPSS 17.0 software. The data were expressed as the mean value and standard deviation. Statistical significance was evaluated via one-way analysis of variance (ANOVA).

## Results

### Growth Characteristics of ‘O’Neal’ Hypanthium and Fruit Throughout Development

The flower bud enlargement process of southern highbush blueberry cultivar ‘O’Neal’ could be divided into 5 stages (I∼V) as described previously (Dernisky et al., 2005), while developing fruits were separated into six stages (S1∼S6, Figure 1B) following its double-sigmoidal growth pattern, in which the fully developed flowers (anthesis) were defined as stage S0 (Yang et al., 2018). A steep rise in the mass of fruit from stages S2 to S6 was accompanied by an increase in the horizontal and vertical diameters (Figure 1C). Pigments were initially accumulated in the peel beginning at stage S5, and fruit gradually became purple or blue and matured at stage S6.

According to microscopic assay of ‘O’Neal’ hypanthia and fruits, the equatorial hypanthium/fruit area maintained a moderate increase during ‘O’Neal’ flower bud enlargement, and accelerated expansion after pollination and fertilization (Figure 1D). The total cell numbers of equatorial hypanthia and fruits throughout development increased by approximately 4,600 (∼3,600 cells before anthesis and ∼1,000 cells after anthesis), whereas the mean cell volume increased approximately 17 fold after anthesis, but only expanded ∼4.5 fold during flower bud enlargement (Figure 1E). In line with the flower and fruit growth indexes and anatomical features, the 12 flower bud and fruit developmental stages of ‘O’Neal’ could be categorized into 3 phases, the cell division phase (stages I∼V), the arrest phase (stages S0∼S1) and, the cell expansion phase (stages S2∼S6).

### Temporal Changes in Physiological Indicators, and Endogenous IAA and ABA Levels of ‘O’Neal’ Hypanthium and Fruit Throughout Development

**FIGURE 2:**
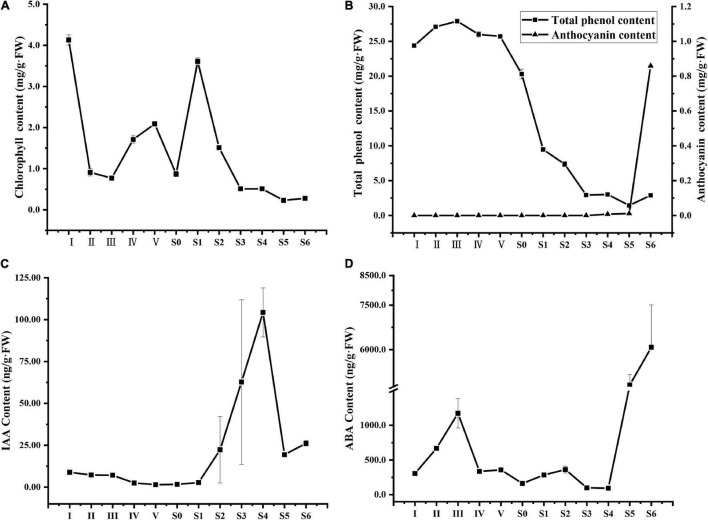
Changes in the chlorophyll **(A)**, total phenol and anthocyanin **(B)**, IAA **(C)** and ABA **(D)** contents of *V. corymbosum* ‘O’Neal’ flower bud and fruit throughout development. Error bars represented the SD of three-to-six independent replicates.

As shown in Figure 2A, the chlorophyll levels of ‘O’Neal’ hypanthia rapidly decreased at stages I∼II and then gradually increased and decreased during later flower bud enlargement. After pollination and fertilization (stages S0∼S1), chlorophyll levels promptly decreased and became undetectable. The content of total phenolic compounds was maintained at a high level during flower bud enlargement (stages I∼IV), decreased sharply beginning at anthesis (stage S0), and slightly increased at stage S5 (Figure 2B). Conversely, anthocyanins were undetectable before stage S3 but began accumulating at stage S4 (approximate 0.0076 mg/g.FW), and reached the highest level in mature fruit (approximate 0.95 mg/g.FW, Figure 2B).

The endogenous levels of phytohormones IAA and ABA during the entire development of flowers bud and fruit were also investigated. Endogenous IAA maintained relatively low levels at the phases of cell division and arrest, while a sharp increase was observed through stages S2 to S4, and a sudden decrease occurred at the rapid accumulation stage of anthocyanins (stage S5, Figure 2C). Similarly, the ABA levels of hypanthia and young fruits were low and, increased significantly during maturation (Figure 2D).

### Transcriptomic Analysis of ‘O’Neal’ During Flower Bud and Fruit Development

According to previous study (Yang et al., 2021), the cell number and area of outer mesocarp and columella at the stages of III∼IV, S0∼S2 and S4∼S5 were significant different during floral and fruit development between large-size cultivar ‘O’Neal’ and small-size cultivar ‘Bluerain,’ indicating these 8 specific stages we chose were more important for southern highbush blueberry fruit growth and development. To understand the regulatory mechanisms of ‘O’Neal’ flower bud and fruit development, especially for fruit size/weight, transcriptomic data were generated by high-throughput RNA-seq. Twenty-four cDNA libraries were constructed from samples of 8 specific developmental stages with three biological replicates. The basic characteristics of the 24 transcriptomic datasets were provided in Supplementary Table 2. In total, 191.61 Gb of clean data was filtered, and 6.32∼10.54 Gb of clean data with a Q30 value higher than 91% was obtained for each sample. High mapping rates (89.96∼91.34%) mapped to the ‘Draper’ reference genome were obtained, and unique mapped rates were 56.73∼61.29%. The expression values, indicated by FPKM, showed positive correlations (Spearman correlation coefficient = 0.74∼0.84) among biological replicates (Supplementary Figure 1). However, the FPKM values of most transcripts (> 50%) were lower than 0.5 (Supplementary Table 3). Interestingly, the ratios of expressed transcripts with low abundance (FPKM values < 0.5) and high abundance (FPKM values > 20) at mature stages (stages S5 and S6) were significantly different from those of other samples (Supplementary Table 3).

**FIGURE 3:**
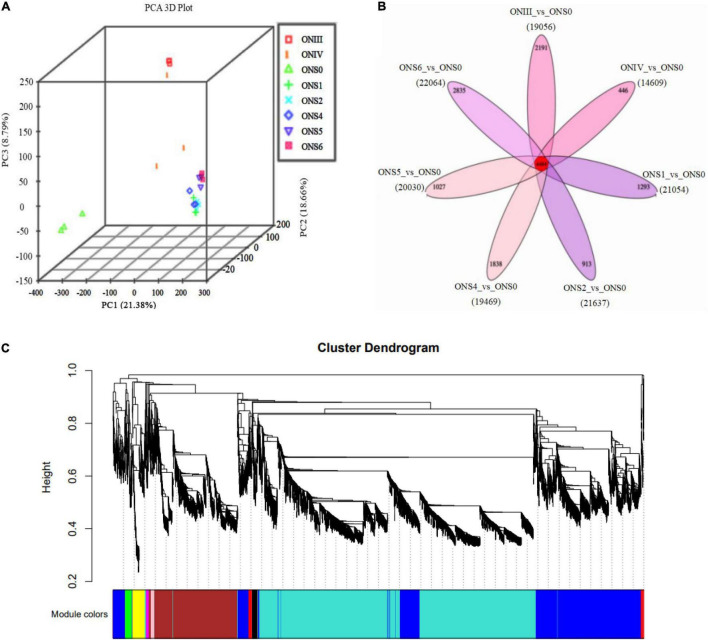
Developmental transcriptomic profiling of *V. corymbosum* ‘O’Neal’ flower bud and fruit. **(A)** Principal component analysis (PCA) of transcriptomic profiles from 8 specific developmental stages. **(B)** Venn diagram illustrating the number of differentially expressed genes (DEGs) shared by or specific to flower bud and fruit development. **(C)** Hierarchical cluster dendrogram related to the cell numbers of outer mesocarp and columella identified by weighted gene co-expression network analysis (WGCNA).

Principal component analysis was performed to detect major trends in transcriptomic data and to visualize similarities among the 8 stages of ‘O’Neal’ flower bud and fruit development. With the exception of the ONIV stage, all biological replicates clustered closely together (Figure 3A). In addition, the fruit samples (stages S1∼S6) all clustered closely together and separately from the flower bud and anthesis stages, suggesting that the developmental processes of blueberry flower buds and fruits were significantly different. Of the expressed transcripts, almost one quarter DEGs were expressed ubiquitously among the samples (Figure 3B).

Since the floral and young fruit phases (before and after anthesis) were the important stages for blueberry cell proliferation and cell expansion initiation, respectively (Yang et al., 2021), the transcriptomic data of samples at stage ONS0 (growth arrest period) as comparison standards, and DEGs at different developmental stages were analyzed (Table 1). With the exception of 14,609 DEGs from the ONIV *vs*. ONS0 comparison, the number of DEGs exceeded 19,000. All DEGs in the 24 samples were then visualized by a Venn diagram (Figure 3B), and 2,191, 446, 1,293, 913, 1,838, 1,027, 2,835 DEGs were specifically expressed at stages III, IV, S1, S2, S4, S5, and S6, respectively. To investigate differences in the functional and metabolic pathways during ‘O’Neal’ flower bud and fruit development, KEGG enrichment analysis was performed, and the pathways with a Padj. value lower than 0.5 were shown in Supplementary Table 4. The annotated DEGs detected for all 7 comparisons shared were highly enriched in the pathways of “biosynthesis of secondary metabolites (ko01110)” and “plant hormone signal transduction (ko04075).” Similarly, numerous DEGs shared among the 7 comparisons were enriched in 154 GO pathways (Supplementary Figure 2), including “hormone-mediated signaling pathway (GO: 0009755)” and “response to abscisic acid (GO: 0009737).”

**TABLE 1:** The numbers of differentially expressed genes (DEGs, Log_2_FC > 1.58 and FDR < 0.01) throughout ‘O’Neal’ flower bud and fruit development for each comparison.

Comparisons	Upregulated DEGs	Downregulated DEGs	Total DEGs
ONIII *vs*. ONS0	10,039	9,017	19,056
ONIV *vs.* ONS0	8,833	5,776	14,609
ONS1 *vs.* ONS0	9,196	11,858	21,054
ONS2 *vs.* ONS0	9,515	12,122	21,637
ONS4 *vs.* ONS0	8,914	10,555	19,469
ONS5 *vs.* ONS0	10,841	9,189	20,030
ONS6 *vs.* ONS0	12,146	9,918	22,064

*ONIII, ONIV, ONS0, ONS1, ONS2, ONS4, ONS5, and ONS6 represented the samples collected from corresponding developmental stages. FC indicated fold change; FDR indicated false discovery rate.*

### Co-expression Networks Associated With Paralogous DEGs and Cell Number Proliferation Patterns of ‘O’Neal’ Outer Mesocarp and Columella

**FIGURE 4:**
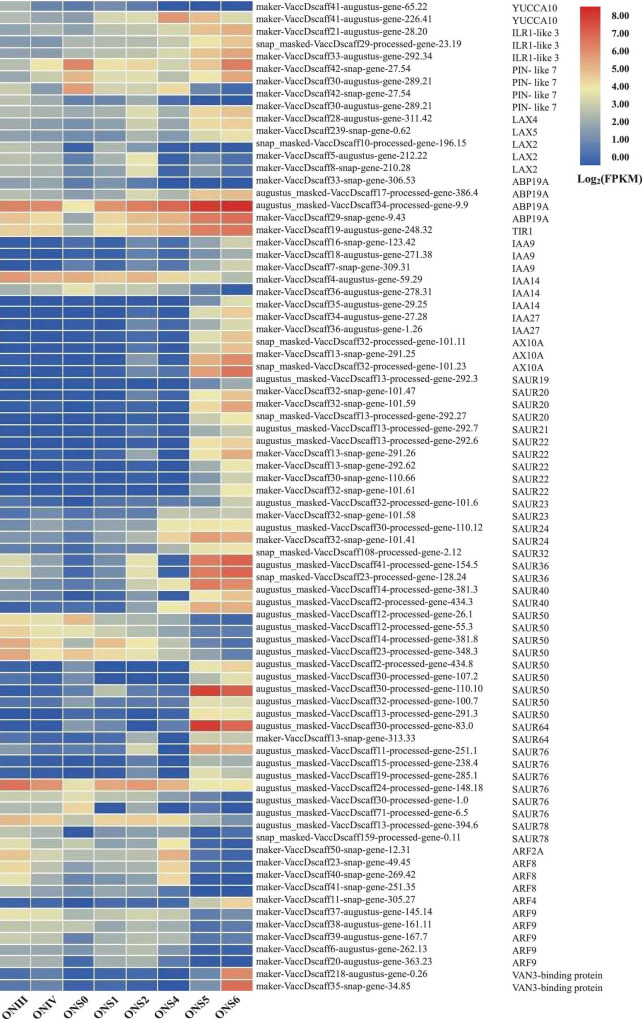
Developmental expression patterns of auxin-related DEGs associated with cell number changes of *V. corymbosum* ‘O’Neal’ outer mesocarp and columella. Each stage of flower bud and fruit development in ‘O’Neal’ was listed horizontally. The color represented the expression levels (Log_2_FPKM) of DEGs.

WGCNA is a popular method developed to explore and highlight gene expression sets with context differences and traits (Cantor and Cordell, 2016). Here, a total of 107,702 transcripts were generated to discover the correlation of ‘O’Neal’ flower bud and fruit development with the gene expression patterns and cell numbers of outer mesocarp and columella. After excluding outlier samples (ONS0 and ONS4), similar expression patterns were characterized with a topological overlap matrix by using the hierarchical clustering method and the dynamic tree cut module (Figure 3C). Ten valid modules were generated, and 5,081 DEGs in the turquoise module were positively or negatively correlated with cell numbers of outer mesocarp (0.94, *p* < 0.005) and columella (−0.94, *p* < 0.005), respectively (Supplementary Table 5), indicating that these DEGs possibly involved in the cell number changes of outer mesocarp and columella, as well as blueberry fruit size variation. Strangely, only a few DEGs involved in “cell cycle (GO:0007049)” and “cell division (GO:0051301)” pathways, such as *cell division control protein 2 homolog C* (*CDC2C*) and *proliferating cell nuclear antigen* (*PCNA*), were associated with the turquoise module. On the contrary, several DEGs in the turquoise module were identified as being involved in auxin and ABA biosynthesis, transportation, signal recognition and transduction, including auxin-activated signaling pathway (GO: 0009734), auxin metabolic process (GO: 0009850), positive regulation of auxin mediated signaling pathway (GO: 0010929), and abscisic acid biosynthetic process (GO: 0009688), *etc*. (Supplementary Table 6). Furthermore, the transcript abundance of auxin-related DEGs in the turquoise module showed two distinctive patterns (Figure 4). Specifically, the expression abundance of *IAA amino acid hydrolase ILR1-like* genes (*LAX4* and *LAX5*), *auxin-binding protein 19a* (*ABP19a*), *auxin/indole-3-acetic acid* members (*IAA9* and *IAA27*), and 4 *small auxin upregulated RNA 50* (*SAUR50*) genes generally decreased with ‘O’Neal’ flower bud and fruit growth and development, whereas that of the others progressively increased, especially during fruit maturation (stages S5 and S6).

### Relative Expression Levels of Auxin Related Genes

**FIGURE 5:**
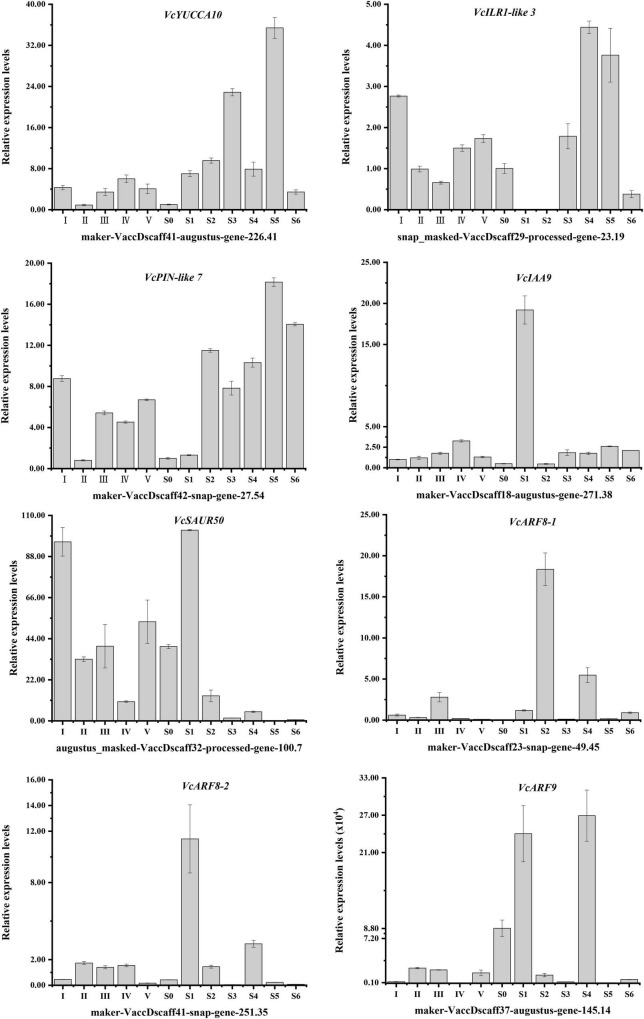
Expression patterns of specific auxin-related DEGs throughout *V. corymbosum* ‘O’Neal’ flower bud and fruit development.

To verify transcriptomic data accuracy, as well as to obtain the detailed expression profiles with hypanthia/fruits developmental dynamics, the relative expression levels of eight auxin biosynthesis-, transportation- and signal transduction-related genes with higher FPKM values were shown in Figure 5. The relative expression patterns of these genes were generally consistent with the transcriptomic data. In addition to the reference gene *VcGAPDH*, *VcIAA9* transcripts at stage I were chosen to normalize the relative expression levels of other auxin-related genes. Of these genes, *VcSAUR50* (*gene-100.7*) showed the highest expression level during flower bud enlargement and early fruit development, but its transcripts decreased rapidly followed by auxin accumulation. Conversely, the transcript levels of *VcILR1-like 3*, *VcIAA9*, *VcARF8-1* and *VcARF8-2* were relatively low during most developmental stages. The relative expression patterns of these auxin-related genes were generally consistent with the transcriptomic data and exhibited increasing (*VcYUCCA10*, *VcPIN-like 7* and *VcARF9*) or decreasing (*VcIAA9* and *VcARF8*-2) trends with the progression of flower and fruit development, indicating these genes involved in auxin accumulation, transportation and responses.

## Discussion

The southern highbush blueberry cultivar ‘O’Neal’, bred in North Carolina in 1987, is an excellent cultivar with low chilling requirement, self-fertility, early ripening, good flavor, small picking scar, and large fruits (Retamales and Hancock, 2018). Similar to other berries, ‘O’Neal’ berries initiate growth after successful pollination and fertilization, and expand quickly by cell division, cell expansion, and accumulation of water and assimilates with seed development in several weeks. During fruit growth and development, the transient concentrations of phytohormones may regulate fruit set, development, and maturation as well as cell proliferation, growth and death (Kumar et al., 2014; Wang et al., 2018; Fenn and Giovannoni, 2021). In this study, cytological, physiological and transcriptomic datasets were used to elucidate the possible regulatory mechanisms underlying flower and fruit development in the southern highbush blueberry cultivar ‘O’Neal’.

### ‘O’Neal’ Hypanthium and Fruit Exhibited a Distinctive Cell Proliferation Pattern Throughout Development

Similar to that of other tissues and organs, the growth of ‘O’Neal’ hypanthium and fruit tissues is mainly determined by cell number and cell expansion. In fact, most fruits resume growth followed by an active cell division phase, and a long cell expansion phase with a long/short period of coexistence and overlap (Dash et al., 2013; Renaudin et al., 2017; Liao et al., 2018; Mauxion et al., 2021). For instance, tomato pericarp cells divided and expanded at similar rates up to 9 days after anthesis, and expanded continually for up to 3∼4 more weeks. During the cell division phase, new cell layers in the tomato pericarp originated from periclinal divisions in the two outer layers (Renaudin et al., 2017; Mauxion et al., 2021). However, the cell proliferation pattern of ‘O’Neal’ fruit was differentiated from the model fruit growth pattern. ‘O’Neal’ outer mesocarp exhibited the highest mitotic activity throughout development, whereas the cell number of columella decreased dramatically (Yang et al., 2021). This may explain why approximately 2/3 of new ‘O’Neal’ fruit cells were produced before anthesis, but the total cell number remained constant throughout fruit development until maturation. In addition, our prior studies measuring equatorial and vertical cell layers (Yang et al., 2019, 2021) indicated that anticlinal and periclinal divisions simultaneously occurred during blueberry flower bud enlargement and the second rapid fruit growth phase, but were restrained at the arrest phase.

### Auxin Promoted ‘O’Neal’ Flower Bud and Fruit Development Through Special Pathways

Phytohormones are a class of small molecular compounds that coordinate responses to developmental programs and environmental changes, and are fundamental for agronomic yield (Krouk et al., 2011; McAtee et al., 2013; Kumar et al., 2014; Liao et al., 2018; Fenn and Giovannoni, 2021). As the earliest discovered plant endogenous phytohormone, auxin integrates with gibberellins and cytokinins, and spatially and temporally controls considerable aspects of fruit development, including fruit set, growth, maturation, and ripening (Pattison et al., 2014; Fenn and Giovannoni, 2021). High-level IAA in the tomato ovary regulates rapid cell division during early exponential growth, and then an internal-to-external IAA gradient pattern appears at the cell expansion stage, while the IAA levels decrease and remain undetectable in the placenta and pericarp (Pattison and Catalá, 2012; McAtee et al., 2013; Fenn and Giovannoni, 2021). Nevertheless, the IAA concentration in the ‘O’Neal’ hypanthium maintained a relatively low level, rapidly increased beginning in the early cell expansion phase, and reached a relatively high level during maturation (Figure 2C). Bu et al. (2020) reported that higher endogenous IAA was synthesized by a larger apple cultivar, and exogenous 1-naphthylacetic acid (NAA) treatment significantly increased fruit mass, whereas 2,3,5-triiodobenzoic acid (TIBA, an inhibitor of auxin polarity transportation) significantly decreased fruit mass. Therefore, high-level endogenous auxin in blueberry fruits might promote fruit cell expansion (not cell proliferation) and result in the production of larger fruits.

Some early auxin responsive members, such as Aux/IAAs, SAURs, and auxin response factors (ARFs), have been clearly shown to regulate fruit set, growth and development (Pandolfini et al., 2007; Kumar et al., 2014; Pattison et al., 2014; Hou et al., 2017, 2020; Luo et al., 2018; Fenn and Giovannoni, 2021; Zong et al., 2021). Low-level auxin promotes the binding of short-lived Aux/IAAs to ARFs and suppresses ARF activities. Conversely, Aux/IAAs are ubiquitinated and degraded under conditions of high auxin levels, and the released ARFs bind to AuxRE regions of target gene promoters, which activate or repress auxin-driven responses (Pandolfini et al., 2007; Fenn and Giovannoni, 2021). To our surprise, only a few early auxin responsive genes were included in the valid WGCNA clustering. Furthermore, accompanying with hypanthium/fruit growth and auxin accumulation, the transcript abundances of *VcSAUR50* (*gene-100.7*) decreased, and the transcript abundances of *VcIAA9* (*gene-271.38*) were almost constant and significantly lower than those of other auxin-related DEGs in the turquoise module, which differed from other *VcSAUR50* and *VcIAA9* members (Figures 4, 5), indicating that *VcIAA9* (*gene-271.38*) and *VcSAUR50* (*gene-100.7*) might be insensitive to auxin, and play a special role in the growth and development process of southern highbush blueberry fruits.

### ABA Was the Main Contributor to ‘O’Neal’ Fruit Maturation and Ripening

Ethylene and ABA are both primary phytohormones that regulate the maturation and ripening processes of climacteric and non-climacteric fruits (Zifkin et al., 2012; McAtee et al., 2013; Fenn and Giovannoni, 2021). Because only a weak peak of respiration and ethylene production has been reported during blueberry maturation (Windus et al., 1976; Suzuki et al., 1997), ABA is believed to be the key phytohormone of ripening processes and pigment accumulation, and to respond to internal and external environmental changes (Kumar et al., 2014; Liao et al., 2018; Han et al., 2021; Kou et al., 2021). During fruit ripening, the firmness of ‘O’Neal’ fruits decreased rapidly beginning at stage S5 (unpublished data), and the cell walls of middle mesocarp collapsed (Yang et al., 2021). Meanwhile, ABA and anthocyanin began to accumulate rapidly (Figure 2). Zifkin et al. (2012) reported that free ABA markedly increased from the ripening transition and was mainly distributed in the fleshy tissues of blueberry fruits. These results indicated that stage S5 was the transition stage for blueberry fruit maturation and ripening, and might integrate or crosstalk with sugar, transcription factors and other phytohormones to regulate complex fruit maturation and ripening processes, as has been observed in other systems such as tomato, strawberry (Kumar et al., 2014; Quinet et al., 2019; Fenn and Giovannoni, 2021). However, ABA was mostly localized in tomato seeds, transported to the surrounding fleshy tissues, and reached a peak at the stage of cell enlargement (Gillaspy et al., 1993). In addition, ABA-deficient tomato mutants produced smaller fruits (Nitsch et al., 2012). The evidence suggested that ABA might regulate fruit maturation and ripening via different pathways between climacteric and non-climacteric fruits (Gillaspy et al., 1993; Zifkin et al., 2012; Yang et al., 2021), and more research is required to elucidate the underlying molecular circuits.

**FIGURE 6:**
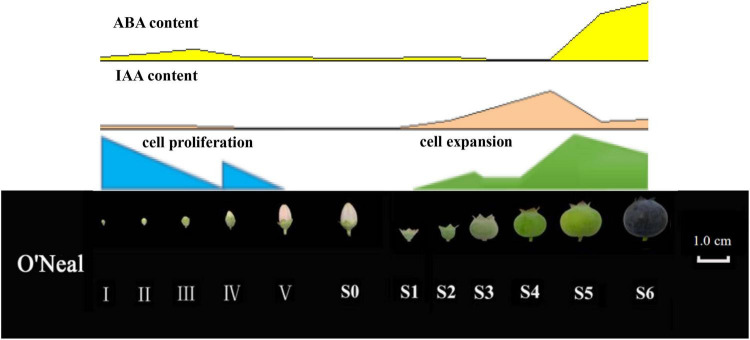
Morphological, cytological and phytohormonal changes of *V. corymbosum* ‘O’Neal’ throughout development.

Overall, time-course cytological, physiological and transcriptomic changes throughout flower and fruit ontogeny in the southern highbush blueberry cultivar ‘O’Neal’ were identified. ‘O’Neal’ hypanthium and fruit exhibited a distinctive cell proliferation pattern, and approximately 2/3 of new cells were produced before anthesis, while the mean cell volume increased approximately 17 fold after anthesis followed with auxin and ABA accumulation (Figure 6). Transcriptomic analysis identified several auxin-related DEGs positively or negatively correlated with cell numbers of key tissues determining fruit size variation. The results suggested that auxin and ABA are key regulatory factors associated with cell proliferation, expansion and fruit maturation. Therefore, the results of this study have established a conceptual framework for blueberry flower and fruit development, which will facilitate additional mechanistic basis for regulating blueberry flower bud enlargement, fruit set and growth, and increasing blueberry fruit quality and production.

## Data Availability Statement

The original contributions presented in the study are publicly available. This data can be found here: The raw transcriptomic data underlying this article were uploaded to the sequence read archive database (SRA, http://www.ncbi.nlm.nih.gov/sra/; BioProject IDs: PRJNA691461, PRJNA692024, PRJNA692267, PRJNA692639, PRJNA695082, and PRJNA773973).

## Author Contributions

LYa and WG conceived and designed the study. LL and YZh performed most of the experiments, with assistance from SF, LYu, YL, YZo, and FL. LL and LYa wrote the manuscript. All authors contributed to the article and approved the submitted version.

## Conflict of Interest

The authors declare that the research was conducted in the absence of any commercial or financial relationships that could be construed as a potential conflict of interest.

## Publisher’s Note

All claims expressed in this article are solely those of the authors and do not necessarily represent those of their affiliated organizations, or those of the publisher, the editors and the reviewers. Any product that may be evaluated in this article, or claim that may be made by its manufacturer, is not guaranteed or endorsed by the publisher.

## Footnotes

1
http://bioinformatics.psb.ugent.be/webtools/Venn/
