# Supplementary material for: Transcriptomic and Physiological Analysis Reveals the Responses to Auxin and Abscisic Acid Accumulation During Vaccinium corymbosum Flower Bud and Fruit Development
Source: Front Plant Sci. 2022 Feb 15;13:818233. doi: 10.3389/fpls.2022.818233 (PMC8886112; doi:10.3389/fpls.2022.818233)
Supplement: Supplementary file 1 [file Data_Sheet_1.PDF]

frontiers

Supplementary Material

Legends of Supplementary Tables and Figures

Table S1. Primers used in this study.

Table S2. Transcriptomic quality and alignment statistics of *V. corymbosum* 'O'Neal' flower bud and fruit throughout development.

Notes: ONIII, ONIV, ONS0, ONS1, ONS2, ONS4, ONS5 and ONS6 represented the samples collected from corresponding developmental stages; "_1", "_2" and "_3" represented the replicates.

Table S3. Expressed gene distribution of each stage during *V. corymbosum* 'O'Neal' flower bud and fruit development.

Note: Expressed gene numbers per category were determined according to FPKM values.

Table S4. KEGG functional classification of DEGs for each comparison (Padj. < 0.5) during *V. corymbosum* 'O'Neal' flower bud and fruit development.

Table S5. Association of gene co-expression modules with developmental stages and cell numbers of outer mesocarp and columella.

Notes: The first number in each cell represents the correlation value, and the second number in the parenthesis represents the p-value of the correlation.

Table S6. GO functional classification of DEGs from the turquoise module (Padj. < 0.04).

Fig. S1. Spearman correlation coefficients of transcriptomic profiles for *V. corymbosum* 'O'Neal' flower bud and fruit.

Fig. S2. GO functional classification of DEGs for each comparison during *V. corymbosum* 'O'Neal' flower bud and fruit development.

Table S1 Primers used in this study.

Gene name	Primer sequences (5'-3')	Predicted Lenth (nt)	Tm (℃)
VcGAPDH (reference gene)	F：TGAGAAAGAATACAAGCCAGAT<br>R：CAGGCAACACCTTACCAA	80	60
VcYUCCA10 (maker-VaccDscaff41-augustus-gene-226.41)	F：TTGCGTAGATGGGACATGC<br>R：AGTGAGGATGGAGCGAGAG	149	58
VcILR1-like 3 (snap_masked-VaccDscaff29-processed-gene-23.19)	F：CCACTACAACTACATGCTATACGG<br>R：GGGAATATTCAGAAGGGTGCC	114	52
VcPIN-like 7 (maker-VaccDscaff42-snap-gene-27.54)	F：TGATGGCACGACTATAGCAAC<br>R：GAAGCTCGGCATTGAAATATAAGG	140	58
VcIAA9 (maker-VaccDscaff18-augustus-gene-271.38)	F：GAAAGTCGGGGTGGTGTTTC<br>R：GCAAAGCTCGGAATCTCGTT	216	60
VcSAUR50 (augustus_masked-VaccDscaff32-processed-gene-100.7)	F：TCGGAGAGAAGAGGAGCAGA<br>R：GGAAGTGAGAGAGCGAAACAC	158	60
VcARF8 (maker-VaccDscaff23-snap-gene-49.45)	F：ATTAGTGTGTTGAAGACAAGAAAGG<br>R：CGACTTCTCTGTTAGTTGTAGCCG	211	60
VcARF8 (maker-VaccDscaff41-snap-gene-251.35)	F：CAAGCCAACAACCAACCAGA<br>R：GCGAAACGAACCCAGCATAT	201	60
VcARF9 (maker-VaccDscaff37-augustus-gene-145.14)	F：CCCTCTGGTTGATGTGCCTA<br>R：CACGGATCAGGACTGGTAGG	146	60

Table S2 Transcriptomic quality and alignment statistics of *V. corymbosum* 'O'Neal' flower bud and fruit throughout development.

Samples	Raw Reads	Clean Reads	Clean Bases (Gb)	Error rate (%)	Q30 (%)	GC Content (%)	Reads aligned onto the 'Draper' genome	
							Total mapped	Unique mapped
ONIII_1	49,139,922	49,119,264	7.34	0.026	93.29	46.61	44,806,062 (91.22%)	29,326,349 (59.70%)
NIII_2	56,700,954	56,675,314	8.47	0.026	93.27	46.46	51,552,675 (90.96%)	33,432,716 (58.99%)
ONIII_3	53,219,882	53,196,442	7.95	0.026	93.06	46.66	48,451,448 (91.08%)	31,417,817 (59.06%)
ONIV_1	54,947,582	54,921,642	8.20	0.026	93.16	46.48	49,819,744 (90.71%)	32,042,904 (58.34%)
ONIV_2	70,539,042	70,507,736	10.54	0.026	92.97	46.39	64,291,603 (91.18%)	41,430,933 (58.76%)
ONIV_3	51,654,142	51,626,838	7.71	0.027	92.66	46.51	46,707,248 (90.47%)	29,786,497 (57.70%)
ONS0_1	52,957,444	52,933,156	7.91	0.026	93.16	46.31	48,330,011 (91.30%)	30,274,073 (57.19%)
ONS0_2	65,342,744	65,319,356	9.75	0.027	92.85	46.06	59,455,237 (91.02%)	37,054,717 (56.73%)
ONS0_3	53,545,034	53,525,490	7.99	0.026	93.31	46.00	48,830,623 (91.23%)	30,609,667 (57.19%)
ONS1_1	57,187,454	57,168,880	8.54	0.026	93.33	46.14	52,069,026 (91.08%)	33,600,433 (58.77%)
ONS1_2	54,173,488	54,153,208	8.09	0.026	93.09	46.13	49,364,616 (91.16%)	32,029,455 (59.15%)
ONS1_3	55,772,360	55,743,750	8.32	0.026	93.11	46.12	50,683,748 (90.92%)	32,763,840 (58.78%)
ONS2_1	60,523,710	60,496,090	9.04	0.027	92.85	45.84	54,788,025 (90.57%)	36,080,316 (59.64%)
ONS2_2	55,478,568	55,459,114	8.29	0.027	92.54	46.23	50,354,257 (90.80%)	32,683,589 (58.93%)
ONS2_3	56,723,832	56,702,582	8.48	0.026	93.09	46.18	51,705,806 (91.19%)	33,886,819 (59.76%)
ONS4_1	56,967,558	56,937,588	8.51	0.029	91.04	47.20	51,370,977 (90.22%)	34,781,283 (61.09%)
ONS4_2	48,640,148	48,623,290	7.27	0.026	93.29	47.36	44,454,532 (91.43%)	29,801,027 (61.29%)
ONS4_3	48,590,412	48,568,764	7.26	0.027	92.97	47.59	44,362,013 (91.34%)	29,682,395 (61.11%)
ONS5_1	56,351,294	56,323,732	8.41	0.027	92.36	46.74	50,951,367 (90.46%)	33,974,793 (60.32%)
ONS5_2	50,325,608	50,303,110	7.52	0.027	92.55	46.81	45,748,598 (90.95%)	30,715,838 (61.06%)
ONS5_3	42,353,968	42,331,934	6.32	0.027	92.90	46.59	38,403,573 (90.72%)	25,757,472 (60.85%)
ONS6_1	49,707,840	49,689,724	7.43	0.026	93.02	46.75	45,203,779 (90.97%)	30,666,664 (61.72%)

ONS6_2	59,658,836	59,640,076	8.92	0.027	92.74	46.49	54,371,698 (91.17%)	36,609,378 (61.38%)
ONS6_3	55,932,558	55,901,724	8.35	0.027	92.87	46.87	50,291,320 (89.96%)	33,654,915 (60.20%)

Notes: ONIII, ONIV, ONS0, ONS1, ONS2, ONS4, ONS5 and ONS6 represented the samples collected from corresponding developmental stages; "_1", "_2" and "_3" represented the replicates.

Table S3 Expressed gene distribution of each stage during *V. corymbosum* 'O'Neal' flower bud and fruit development.

Samples	0.5 ≤ FPKM < 2	2 ≤ FPKM < 20	20 ≤ FPKM < 50	50 ≤ FPKM
ONIII	11,591 (17.69%)	15,930 (24.31%)	1,728 (2.64%)	975 (1.49%)
ONIV	12,181 (18.59%)	16,886 (25.77%)	1,785 (2.72%)	1,038 (1.58%)
ONS0	10,453 (15.95%)	14,943 (22.80%)	2,180 (3.32%)	1,288 (1.97%)
ONS1	11,232 (17.14%)	15,985 (24.39%)	1,956 (2.98%)	1,137 (1.73%)
ONS2	11,259 (17.18%)	16,305 (24.88%)	1,892 (2.89%)	1,064 (1.62%)
ONS4	11,376 (17.36%)	16,290 (24.86%)	1,840 (2.81%)	1,115 (1.70%)
ONS5	9,591 (14.63%)	14,119 (21.54%)	2,145 (3.27%)	1,489 (2.27%)
ONS6	9,459 (14.33%)	13,587 (20.73%)	2,195 (3.35%)	1,586 (2.42%)

Note: Expressed gene numbers per category were determined according to FPKM values.

Table S4 KEGG functional classification of DEGs for each comparison (Padj. < 0.5) during *V. corymbosum* 'O'Neal' flower bud and fruit development.

**ONIII vs. ONS0**	**ONIV vs. ONS0**
**Biosynthesis of secondary metabolites**	**Biosynthesis of secondary metabolites**
Glycolysis / Gluconeogenesis	MAPK signaling pathway - plant
**Plant hormone signal transduction**	**Plant hormone signal transduction**
Biosynthesis of antibiotics	Glycolysis / Gluconeogenesis
Biosynthesis of amino acids	NOD-like receptor signaling pathway
Methane metabolism	Biosynthesis of antibiotics
Microbial metabolism in diverse environments	Microbial metabolism in diverse environments
MAPK signaling pathway - plant	Carbon metabolism
Carbon metabolism	Glycine, serine and threonine metabolism
Galactose metabolism	Tryptophan metabolism
Tyrosine metabolism	Galactose metabolism
Influenza A	Biosynthesis of amino acids
Hepatitis B	Flavonoid biosynthesis
Starch and sucrose metabolism	Plant-pathogen interaction
	PI3K-Akt signaling pathway
	Phenylpropanoid biosynthesis
	MAPK signaling pathway - fly
	Longevity regulating pathway - multiple species
	Starch and sucrose metabolism
	Ras signaling pathway
	Longevity regulating pathway
	Alcoholism
	Glutamatergic synapse
	Pancreatic cancer
	Pentose and glucuronate interconversions
	Quorum sensing
	Fatty acid metabolism
	Insulin signaling pathway
	Isoquinoline alkaloid biosynthesis
	Antigen processing and presentation

	Measles
	Central carbon metabolism in cancer
	Tyrosine metabolism
	FoxO signaling pathway
	Influenza A
	Rap1 signaling pathway
	VEGF signaling pathway
	Osteoclast differentiation
	Toll-like receptor signaling pathway
	B cell receptor signaling pathway
	Prolactin signaling pathway
	Vasopressin-regulated water reabsorption
	Mineral absorption
	Pertussis
	Metabolic pathways
	Citrate cycle (TCA cycle)
	EGFR tyrosine kinase inhibitor resistance
	Hedgehog signaling pathway - fly
	GABAergic synapse
	Chagas disease (American trypanosomiasis)
	Breast cancer
	Pentose phosphate pathway
	Glutathione metabolism
	Methane metabolism
	Fatty acid degradation
	Thiamine metabolism
**ONS1 vs. ONS0**	**ONS2 vs. ONS0**
**Plant hormone signal transduction**	**Plant hormone signal transduction**
Biosynthesis of amino acids	**Biosynthesis of secondary metabolites**
**Biosynthesis of secondary metabolites**	Glycolysis / Gluconeogenesis
Glycolysis / Gluconeogenesis	Biosynthesis of amino acids
Carbon metabolism	Amino sugar and nucleotide sugar metabolism
Biosynthesis of antibiotics	Glycine, serine and threonine metabolism

Plant-pathogen interaction	
Microbial metabolism in diverse environments	
Influenza A	
MAPK signaling pathway - plant	
**ONS4 vs. ONS0**	**ONS5 vs. ONS0**
Carbon metabolism	**Plant hormone signal transduction**
**Biosynthesis of secondary metabolites**	**Biosynthesis of secondary metabolites**
Microbial metabolism in diverse environments	Glycine, serine and threonine metabolism
Glycine, serine and threonine metabolism	HIF-1 signaling pathway
**Plant hormone signal transduction**	Glycolysis / Gluconeogenesis
Protein processing in endoplasmic reticulum	
Biosynthesis of antibiotics	
Glycolysis / Gluconeogenesis	
Biosynthesis of amino acids	
Plant-pathogen interaction	
Fatty acid metabolism	
Glyoxylate and dicarboxylate metabolism	
Influenza A	
Methane metabolism	
Starch and sucrose metabolism	
TGF-beta signaling pathway	
**ONS6 vs. ONS0**	
**Biosynthesis of secondary metabolites**	
Starch and sucrose metabolism	
**Plant hormone signal transduction**	
Influenza A	
Plant-pathogen interaction	
HIF-1 signaling pathway	
Glyoxylate and dicarboxylate metabolism	
Carbon metabolism	
Fatty acid metabolism	

Phenylpropanoid biosynthesis
Microbial metabolism in diverse environments
Porphyrin and chlorophyll metabolism
Nicotinate and nicotinamide metabolism

Table S5 Association of gene co-expression modules with developmental stages and cell numbers of outer mesocarp and columella.

Modules	Cell numbers of outer mesocarp	Cell numbers of outer columella
MEpink	0.77 (0.08)	-0.66 (0.2)
MEred	0.15 (0.8)	0.04 (0.9)
MEblack	0.69 (0.1)	-0.69 (0.1)
**MEturquoise**	**0.94 (0.005)**	**-0.94 (0.005)**
MEbrown	-0.89 (0.02)	0.59 (0.2)
MEyellow	-0.51 (0.3)	0.29 (0.6)
MEgreen	0.13 (0.8)	0.09 (0.9)
MEblue	-0.48 (0.3)	0.78 (0.07)
MEmagenta	-0.28 (0.6)	0.5 (0.3)
MEgrey	-0.15 (0.8)	0.031 (1.0)

Notes: The first number in each cell represents the correlation value, and the second number in the parenthesis represents the p-value of the correlation.

Table S6 GO functional classification of DEGs from the turquoise module (Padj. < 0.04).

ID	Description	GeneRatio	BgRatio	Padj.
GO:0006949	syncytium formation	24/1971	54/42809	1.87E-15
GO:0016168	chlorophyll binding	24/1971	63/42809	7.00E-14
GO:0009768	photosynthesis_ light harvesting in photosystem I	21/1971	48/42809	1.21E-13
GO:0004420	hydroxymethylglutaryl-CoA reductase (NADPH) activity	12/1971	14/42809	1.30E-12
GO:0080043	quercetin 3-O-glucosyltransferase activity	38/1971	196/42809	6.82E-12
GO:0010360	negative regulation of anion channel activity	11/1971	14/42809	7.24E-11
GO:0018298	protein-chromophore linkage	27/1971	118/42809	3.72E-10
GO:0004721	phosphoprotein phosphatase activity	15/1971	39/42809	6.55E-09
GO:0010344	seed oilbody biogenesis	9/1971	15/42809	2.79E-07
GO:0009734	auxin-activated signaling pathway	45/1971	381/42809	6.32E-07
GO:0008017	microtubule binding	19/1971	95/42809	3.50E-06
GO:0009416	response to light stimulus	26/1971	168/42809	3.50E-06
GO:0042335	cuticle development	11/1971	31/42809	3.50E-06
GO:0006749	glutathione metabolic process	24/1971	147/42809	3.50E-06
GO:0006833	water transport	12/1971	38/42809	3.66E-06
GO:0009664	plant-type cell wall organization	22/1971	131/42809	6.36E-06
GO:0010162	seed dormancy process	14/1971	61/42809	2.24E-05
GO:0009234	menaquinone biosynthetic process	7/1971	14/42809	4.40E-05
GO:0009407	toxin catabolic process	18/1971	104/42809	4.52E-05
GO:0004568	chitinase activity	15/1971	75/42809	4.97E-05
GO:0009269	response to desiccation	7/1971	15/42809	6.78E-05
GO:0015979	photosynthesis	31/1971	271/42809	0.000105
GO:0009718	anthocyanin-containing compound biosynthetic process	17/1971	101/42809	0.000105
GO:0008106	alcohol dehydrogenase (NADP+) activity	6/1971	11/42809	0.000105

GO:0009073	aromatic amino acid family biosynthetic process	14/1971	72/42809	0.000126
GO:0032776	DNA methylation on cytosine	7/1971	17/42809	0.000151
GO:0034220	ion transmembrane transport	11/1971	46/42809	0.000151
GO:0071555	cell wall organization	35/1971	334/42809	0.000151
GO:0071470	cellular response to osmotic stress	6/1971	12/42809	0.000168
GO:0009704	de-etiolation	11/1971	47/42809	0.00017
GO:0042372	phylloquinone biosynthetic process	7/1971	18/42809	0.000201
GO:0060416	response to growth hormone	10/1971	40/42809	0.000218
GO:0015995	chlorophyll biosynthetic process	16/1971	98/42809	0.000218
GO:0019915	lipid storage	9/1971	34/42809	0.00035
GO:0009788	negative regulation of abscisic acid-activated signaling pathway	13/1971	71/42809	0.000387
GO:0042981	regulation of apoptotic process	6/1971	14/42809	0.000405
GO:0009607	response to biotic stimulus	24/1971	204/42809	0.000496
GO:0009765	photosynthesis_ light harvesting	8/1971	28/42809	0.000503
GO:0004860	protein kinase inhibitor activity	7/1971	21/42809	0.000504
GO:0006751	glutathione catabolic process	7/1971	21/42809	0.000504
GO:0006739	NADP metabolic process	6/1971	15/42809	0.000569
GO:0008935	1_4-dihydroxy-2-naphthoyl-CoA synthase activity	5/1971	10/42809	0.000702
GO:1902479	positive regulation of defense response to bacterium_ incompatible interaction	5/1971	10/42809	0.000702
GO:0080148	negative regulation of response to water deprivation	11/1971	57/42809	0.000803
GO:0050826	response to freezing	9/1971	39/42809	0.000867
GO:0009832	plant-type cell wall biogenesis	6/1971	17/42809	0.001159
GO:0009688	abscisic acid biosynthetic process	12/1971	70/42809	0.001173
GO:0009615	response to virus	18/1971	140/42809	0.001195
GO:0033834	kaempferol 3-O-galactosyltransferase activity	6/1971	18/42809	0.001568

GO:0009423	chorismate biosynthetic process	11/1971	62/42809	0.001575
GO:0008428	ribonuclease inhibitor activity	5/1971	12/42809	0.001722
GO:0040008	regulation of growth	23/1971	212/42809	0.001795
GO:0019953	sexual reproduction	6/1971	19/42809	0.002037
GO:0003855	3-dehydroquinate dehydratase activity	5/1971	13/42809	0.002496
GO:0045549	9-cis-epoxycarotenoid dioxygenase activity	5/1971	13/42809	0.002496
GO:0001872	(1->3)-beta-D-glucan binding	6/1971	20/42809	0.0026
GO:0051179	localization	6/1971	20/42809	0.0026
GO:0004097	catechol oxidase activity	7/1971	28/42809	0.002666
GO:0010119	regulation of stomatal movement	12/1971	80/42809	0.003464
GO:0009635	response to herbicide	8/1971	39/42809	0.004018
GO:0005179	hormone activity	7/1971	32/42809	0.006132
GO:0048527	lateral root development	11/1971	74/42809	0.006309
GO:0010224	response to UV-B	17/1971	151/42809	0.006864
GO:0090626	plant epidermis morphogenesis	6/1971	24/42809	0.006864
GO:0009682	induced systemic resistance	8/1971	43/42809	0.007187
GO:0010189	vitamin E biosynthetic process	8/1971	43/42809	0.007187
GO:0007049	cell cycle	14/1971	113/42809	0.007187
GO:0010970	transport along microtubule	4/1971	10/42809	0.007187
GO:0015689	molybdate ion transport	4/1971	10/42809	0.007187
GO:0015810	aspartate transmembrane transport	4/1971	10/42809	0.007187
GO:0015827	tryptophan transport	4/1971	10/42809	0.007187
GO:0033743	peptide-methionine (R)-S-oxide reductase activity	4/1971	10/42809	0.007187
GO:0060271	cilium assembly	4/1971	10/42809	0.007187
GO:2000582	positive regulation of ATP-dependent microtubule motor activity_ plus-end-directed	4/1971	10/42809	0.007187
GO:0004857	enzyme inhibitor activity	7/1971	34/42809	0.007355

GO:0010090	trichome morphogenesis	9/1971	55/42809	0.007941
GO:0015853	adenine transport	4/1971	11/42809	0.010325
GO:0015854	guanine transport	4/1971	11/42809	0.010325
GO:0052793	pectin acetylesterase activity	6/1971	27/42809	0.010864
GO:0005543	phospholipid binding	5/1971	19/42809	0.012151
GO:0010015	root morphogenesis	5/1971	19/42809	0.012151
GO:0042254	ribosome biogenesis	6/1971	28/42809	0.012653
GO:0046355	mannan catabolic process	6/1971	28/42809	0.012653
GO:0016788	hydrolase activity_ acting on ester bonds	9/1971	60/42809	0.0136
GO:0071669	plant-type cell wall organization or biogenesis	5/1971	20/42809	0.014858
GO:0004197	cysteine-type endopeptidase activity	10/1971	73/42809	0.014983
GO:0030570	pectate lyase activity	10/1971	73/42809	0.014983
GO:0004321	fatty-acyl-CoA synthase activity	6/1971	30/42809	0.017
GO:0010053	root epidermal cell differentiation	6/1971	30/42809	0.017
GO:0045735	nutrient reservoir activity	6/1971	30/42809	0.017
GO:0016758	transferase activity_ transferring hexosyl groups	23/1971	264/42809	0.021176
GO:0006782	protoporphyrinogen IX biosynthetic process	7/1971	42/42809	0.021825
GO:0004814	arginine-tRNA ligase activity	4/1971	14/42809	0.02351
GO:0051301	cell division	17/1971	176/42809	0.024659
GO:0009789	positive regulation of abscisic acid-activated signaling pathway	13/1971	119/42809	0.024986
GO:0019632	shikimate metabolic process	5/1971	23/42809	0.025455
GO:0016829	lyase activity	9/1971	67/42809	0.02557
GO:0009845	seed germination	12/1971	107/42809	0.02756
GO:0009695	jasmonic acid biosynthetic process	9/1971	68/42809	0.027719
GO:0010296	prenylcysteine methylesterase activity	4/1971	15/42809	0.028736

GO:0009850	auxin metabolic process	5/1971	24/42809	0.029129
GO:0010507	negative regulation of autophagy	5/1971	24/42809	0.029129
GO:0009055	electron transfer activity	24/1971	296/42809	0.037744
GO:0010929	positive regulation of auxin mediated signaling pathway	6/1971	36/42809	0.037744
GO:0071446	cellular response to salicylic acid stimulus	6/1971	36/42809	0.037744
GO:0031408	oxylipin biosynthetic process	10/1971	85/42809	0.037744
GO:0010143	cutin biosynthetic process	7/1971	48/42809	0.040331
GO:0043547	positive regulation of GTPase activity	6/1971	37/42809	0.042179
GO:0009056	catabolic process	13/1971	129/42809	0.043136
GO:0052689	carboxylic ester hydrolase activity	7/1971	49/42809	0.043989

Fig. S1 Spearman correlation coefficients of transcriptomic profiles for *V. corymbosum* 'O'Neal' flower bud and fruit.

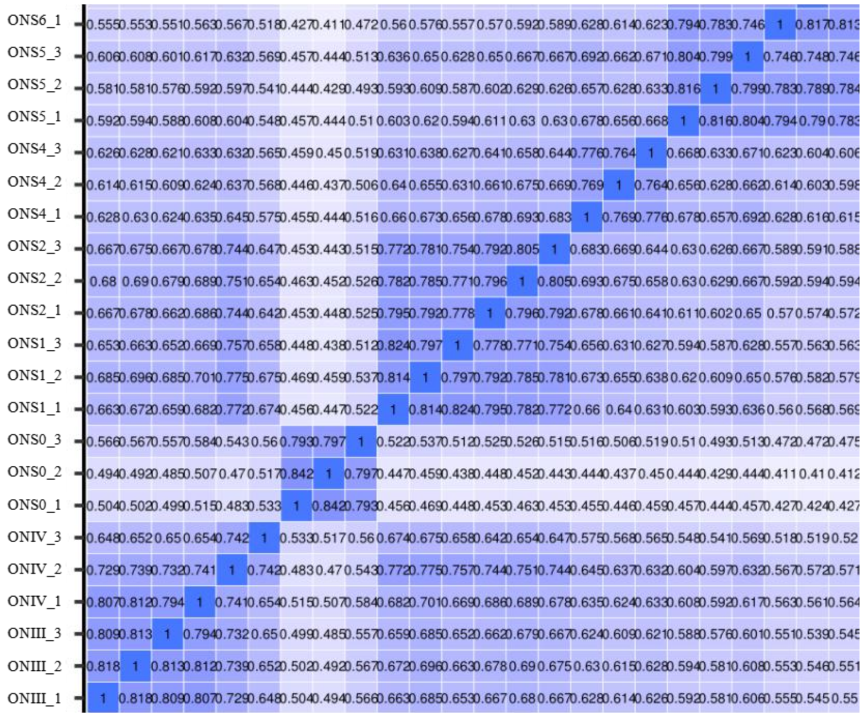

Fig. S2 GO functional classification of DEGs for each comparison during *V. corymbosum* 'O'Neal' flower bud and fruit development.

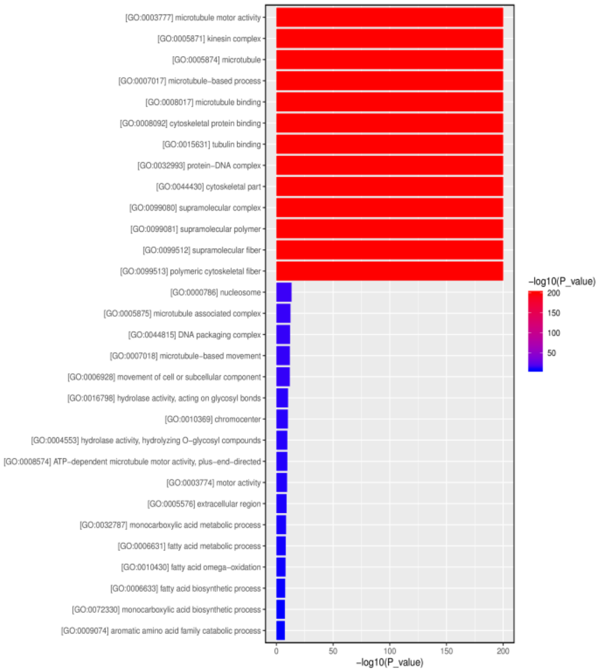

ONIII vs. ONS0

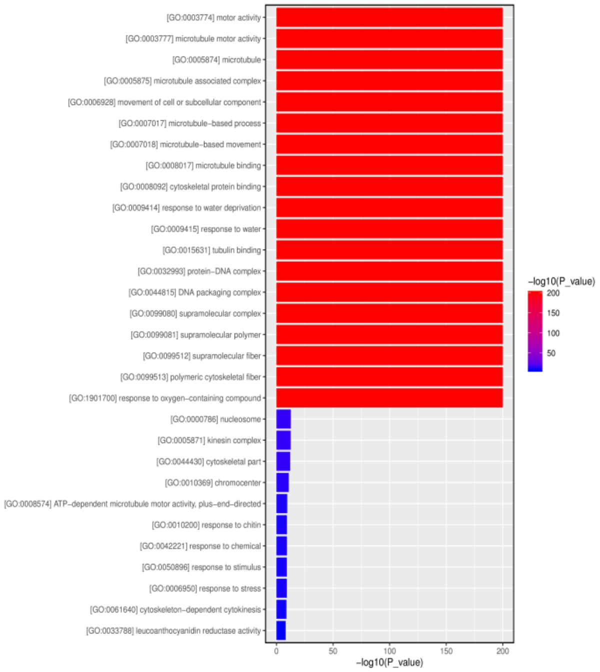

ONIV vs. ONS0

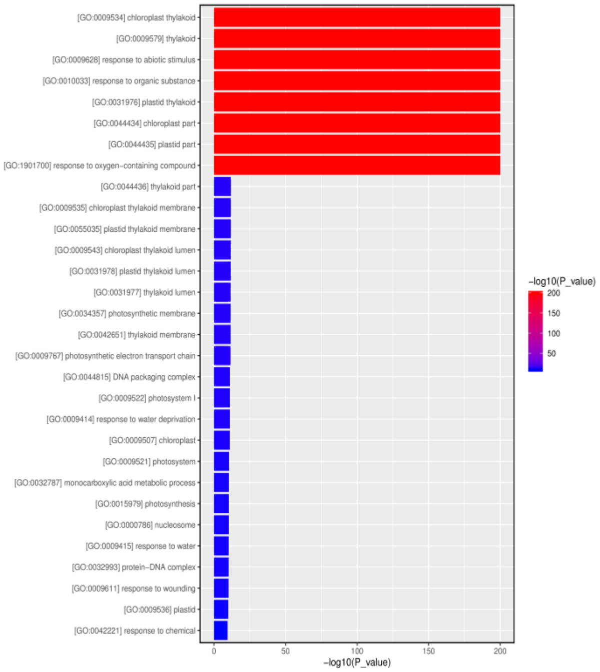

ONS1 vs. ONS0

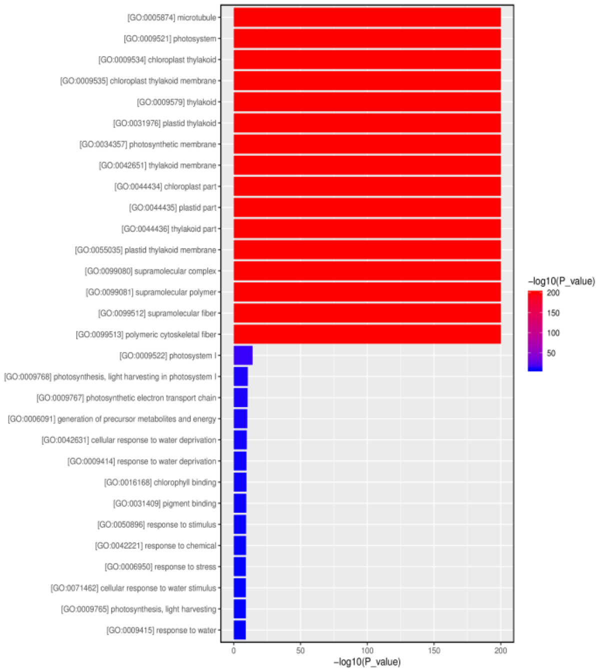

ONS2 vs. ONS0

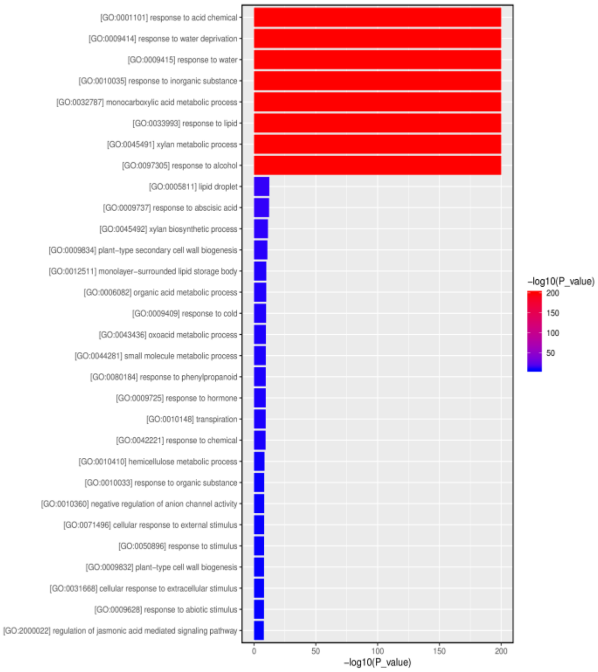

ONS4 vs. ONS0

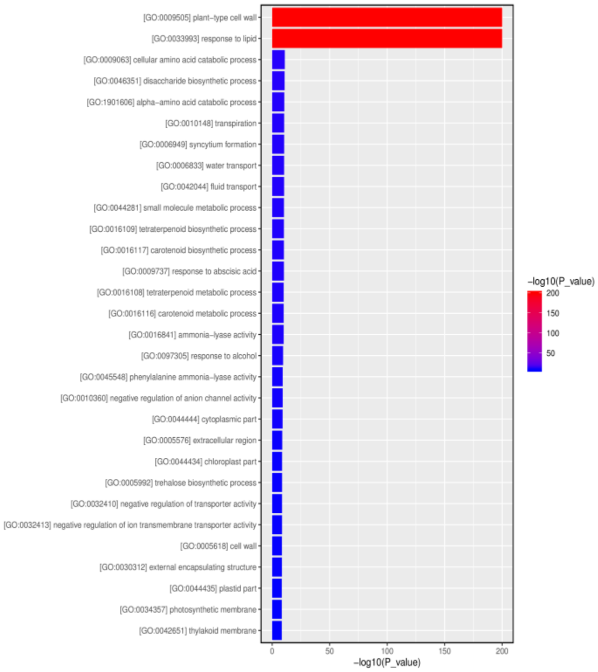

ONS5 vs. ONS0

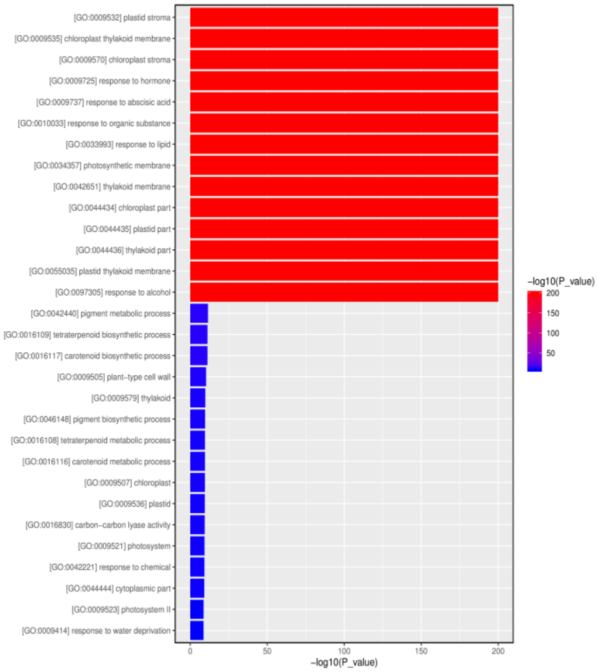

ONS6 vs. ONS0
